# The correlation between neutrophil/lymphocyte ratio and the MRI burden and cognitive function in patients with cerebral small vessel disease

**DOI:** 10.3389/fneur.2025.1546076

**Published:** 2025-05-07

**Authors:** Mengyao Zhao, Xiaohua Xie, Zhiwei Hao, Mengsi Ren, Zhenjie Teng, Jing Xu, Chunhui Shan, Yanhong Dong, Peiyuan Lv

**Affiliations:** ^1^Department of Neurology, Graduate School of Hebei Medical University, Shijiazhuang, China; ^2^Department of Neurology, Hebei General Hospital, Shijiazhuang, China; ^3^Department of Neurology, Graduate School of Hebei North University, Zhangjiakou, China; ^4^Hebei Provincial Key Laboratory of Cerebral Networks and Cognitive Disorders, Shijiazhuang, China; ^5^Department of Radiology, Hebei General Hospital, Shijiazhuang, China

**Keywords:** cerebral small vessel disease (CSVD), cognitive impairment, neutrophil/lymphocyte ratio (NLR), Mini-Mental State Examination (MMSE), total burden

## Abstract

**Objective:**

Aims to explore the relationship between neutrophil/lymphocyte ratio (NLR) and the total burden of imaging markers and cognitive function in patients with cerebral small vessel disease (CSVD).

**Methods:**

A retrospective study was conducted on 148 hospitalized CSVD patients at Hebei General Hospital from January 2022 to September 2024, with complete clinical and laboratory data. NLR was calculated as neutrophil count/lymphocyte count. According to the Mini-Mental State Examination (MMSE) score, patients were divided into a cognitive impairment group (*n* = 89) and a non-cognitive impairment group (*n* = 59). The total CSVD burden was assessed based on magnetic resonance imaging (MRI). We used logistic regression models, restricted cubic spline plots, Spearman correlation, and mediation analysis to evaluate the relationship between NLR in CSVD patients and CSVD burden and cognitive impairment.

**Results:**

The results of the multivariate logistic regression showed that after adjusting for all potential confounding factors, an elevated NLR in CSVD patients was significantly associated with the risk of cognitive impairment (OR: 3.263; 95% CI: 1.577 to 6.752; *p* = 0.001) and severe CSVD burden (OR = 2.246, 95% CI: 1.346 ~ 3.750, *p* = 0.002). The restricted cubic spline plot shows that after adjusting for confounding factors, the NLR level is linearly associated with the risk of CI (*P* for total = 0.022, *P* for non-linear = 0.231) and the total burden of CSVD (*P* for total = 0.005, *P* for non-linear = 0.448). Correlation analysis shows that NLR is positively correlated with the CSVD score (rs = 0.246, *p* = 0.003). Furthermore, the results of the mediation analysis indicate that after adjusting for confounding factors, the burden of CSVD has a significant mediating effect on the relationship between NLR levels and cognitive impairment (ab = 0.028, 95% CI: 0.004 to 0.070, *p* = 0.012); 20.9% of the total effect of NLR on cognitive impairment in CSVD patients can be attributed to the presence of CSVD burden.

**Conclusion:**

Elevated NLR in CSVD patients is associated with the burden of CSVD and cognitive impairment. The mediating role of CSVD burden suggests that elevated NLR may lead to cognitive impairment by exacerbating the burden of CSVD.

## Introduction

With the aging population in our country, the incidence, prevalence, disability rate, and mortality rate of cognitive impairment are increasing year by year. An increasing body of evidence indicates that cerebrovascular lesions are the important triggering factors for dementia, with additive or synergistic effects in relation to neurodegenerative diseases. Dementia has long been considered untreatable, but it can be prevented and even the course might be modifiable by intervening in related risk factors. Cerebrovascular disease is one of the risk factors that can be potentially modifiable (1). Among them, cerebral small vessel disease (CSVD) is one of the important causes of vascular cognitive impairment (2).

Cerebral small vessel disease is a group of various vascular diseases with different pathological types, which can be classified by arteriosclerosis/age-related CSVD, amyloid-related CSVD, genetic CSVD distinct from amyloid angiopathy, inflammatory-immune mediated type, venous collagenosis, and other types (3). Its imaging features include recent small subcortical infarct, white matter hyperintensities (WMHs), lacune of presumed vascular origin, cerebral microbleeds (CMBs), enlarged perivascular space (EPVS), and brain atrophy (4). However, due to the diverse clinical manifestations of patients with CSVD, the potential pathophysiological mechanisms underlying cognitive impairment remain unclear. Moreover, due to the limited understanding of the potential pathophysiology of CSVD and the lack of effective treatment options, early control of risk factors and prevention of further cerebrovascular progression are the fundamental measures to prevent and treat cognitive impairment. Lacunar infarcts is one of the clinical manifestations of CSVD. Studies shown lacunar infarcts are the ischemic stroke subtype with the best functional prognosis, but that the pathophysiology, clinical features and outcome of lacunar strokes are different from other acute ischemic cerebrovascular diseases (5). Therefore, the pathophysiological mechanism of CSVD remains to be further explored.

In recent years, the neutrophil/lymphocyte ratio (NLR), platelet/lymphocyte ratio (PLR), and monocyte/lymphocyte ratio (MLR) have been proposed as newer systemic inflammatory biomarkers. Previous studies have shown that these indicators reflect the inflammatory state induced by various malignant tumors and have been proven to be reliable prognostic tools for different stages of the disease and treatment methods (6–8). In particular, the NLR has been shown to predict early cerebral edema and clinical deterioration after reperfusion therapy for stroke, and it is correlated with early neurological outcomes after thrombolysis in patients with acute ischemic stroke (9), cardiovascular risk in hypertensive patients (10), and cognitive impairment in patients with CSVD (11). However, the relationship between NLR, PLR, and MLR with the total imaging burden and cognitive function in CSVD patients has not been explored simultaneously.

In this study, we explored whether these indicators increase the severity of the burden of CSVD and the risk of cognitive impairment, as well as whether the impact of cognitive impairment is mediated by the severity of the CSVD burden.

## Materials and methods

### Study population

This is a hospital-based retrospective study, selecting patients with CSVD who were hospitalized and completed neuropsychological assessments from January 2020 to September 2024 at Hebei General Hospital. All included subjects had detailed medical histories, physical examinations, relevant laboratory tests, and neuroimaging data. The exclusion criteria were as follows: (1) patients with active infections or antibiotic use within the last 2 weeks; (2) hematological diseases, malignant tumors, autoimmune diseases; (3) recent treatment with immunosuppressants; (4) those with brain tumors or other systemic tumors, surgery, or severe trauma; (5) non-vascular white matter injury, such as metabolic encephalopathy, multiple sclerosis, etc.; (6) cognitive impairment due to other conditions, such as carbon monoxide poisoning, hyperthyroidism, hypothyroidism, severe anxiety, or depression. Ultimately, a total of 148 eligible patients participated in the analysis. CSVD was diagnosed according to the Chinese guidelines for CSVD (12). The interval between CSVD diagnosis and blood tests was within 7 days. This study was conducted according to the declaration of Helsinki and approved by the Ethical Committees of Hebei General Hospital (No.2024-LW-0228).

### Clinical characteristics

All demographic and risk factors were obtained from medical records: age, gender, years of education, body mass index (BMI), smoking status, and alcohol consumption. Medical history was also collected, including hypertension, diabetes, coronary heart disease, and stroke. Laboratory biomarkers were measured, including total cholesterol (TC), triglycerides (TG), high-density lipoprotein cholesterol (HDL-C), low-density lipoprotein cholesterol (LDL-C), total homocysteine (tHcy), white blood cell count, neutrophil count, lymphocyte count, monocyte count, and platelet count. NLR was calculated as neutrophil count/lymphocyte count. PLR was calculated as platelet count/lymphocyte count. MLR was calculated as monocyte count/lymphocyte count. The SII was calculated as platelet count × neutrophil count/lymphocyte count.

### Imaging acquisition and assessment

All eligible participants had completed magnetic resonance imaging (MRI) examination with adequate sequences for the assessment of CSVD. All sequence images were obtained from the medical records. MRI examination was performed in all patients with 3.0-Tesal magnetic resonance scanners (Signa, GE Healthcare of America). The MRI protocol and detailed acquisition parameters are as follows: (1) T1-weighted imaging (T1WI), repetition time (TR)/echo time (TE) = 1909/20.2 milliseconds (ms), field of view (FOV) = 240 × 192 mm^2^, acquisition matrix = 320 × 224, number of excitations (NEX) = 1; (2) T2-weighted imaging (T2WI), TR/TE = 5000/125 ms, FOV = 240 × 240 mm^2^, acquisition matrix = 352 × 352, NEX = 1; (3) Fluid Attenuation Inversion Recovery (FLAIR), TR/TE = 8502/159.4 ms, FOV = 240 × 240 mm^2^, acquisition matrix = 256 × 256, NEX = 1; (4) Sensitivity Weighted Imaging (SWI), TR/TE = 78.6/47.6 ms, FOV = 240 × 216 mm^2^, acquisition matrix = 384 × 320, NEX = 1; (5) Diffusion Weighted Imaging (DWI), TR/TE = 4800/81.7 ms, FOV = 240 × 240 mm^2^, acquisition matrix = 160 × 160, NEX = 1. The slice thickness for SWI is 2 mm, while the slice thickness for T1WI, T2WI, FLAIR, and DWI is 5 mm.

Imaging markers of CSVD (WMHs, CMBs, lacune, EPVS) were evaluated independently by two readers (XX and JX) following the STRIVE criteria (4). In case of disagreement on any markers, a radiologist (CS) was involved to reach a consensus. All ratings were performed blinded to all patient data. The imaging assessment criteria are as follows: WMHs: Manifesting as abnormal signals of varying sizes in the white matter regions, appearing as high signals on T2WI and FLAIR, and as isointense or low signals on T1WI sequences. WMHs are mostly bilaterally symmetrical, primarily distributed around the lateral ventricles or in the deep white matter of the brain. WMHs are assessed using the Fazekas scale: periventricular white matter (PVWMH): 0 points, no lesions; 1 point, lesions appear as cap-like or pencil-thin layers; 2 points, lesions appear as smooth halo-like; 3 points, irregular lesions extend into the deep white matter (DWMH): 0 points, no lesions; 1 point, punctate lesions; 2 points, lesions begin to merge; 3 points, lesions merge extensively (13). Lacune: Presenting as round or oval fluid-filled spaces resembling cerebrospinal fluid signals located in the subcortical area, appearing as low signal on T1WI sequence, high signal on T2WI sequence, and central low signal with a peripheral high signal ring on FLAIR sequence, with a diameter of 3 to 15 mm (4). EPVS: Similar to lacune, presenting as low signal on T1WI and FLAIR sequences, high signal on T2WI sequence, but generally with a diameter of <3 mm, and no high signal ring on FLAIR. EPVS in the basal ganglia were coded according to following scale applied to standard axial images: grade 0 = no EPVS, grade 1 = 1–10 EPVS, grade 2 = 11–20 EPVS and grade 4 = > 40 EPVS. The numbers refer to EPVS on one side of the brain; the higher score was used if there was asymmetry between the sides and EPVS were counted in the slice with the highest number (14). CMBs: Presenting as small round or oval shapes on SWI, with clear boundaries, homogeneity, and signal loss lesions, typically with a diameter of 2–5 mm, and can be up to 10 mm. Among them, deep CMBs (basal ganglia, internal and external capsules, thalamus, corpus callosum, and periventricular white matter) are considered related to small artery atherosclerotic CSVD, thus only the number of deep CMBs is counted (15). CSVD total burden was rated on an ordinal scale from 0 to 4. 1 point is recorded when the following manifestations are present: according to the Fazekas assessment scale, DWMH score ≥ 2 points or (and) PVWMH score of 3 points; deep CMBs ≥ 1; lacune ≥ 1; EPVS grading ≥ 2 (16). The total score is 4 points; a higher score indicates a heavier total burden of CSVD, and the study subjects are divided into a mild burden group (0–2 points) and a severe burden group (3–4 points) based on the total burden (17).

### Neuropsychological assessment

All eligible participants had completed neuropsychological assessments the validated Chinese version of the Mini-Mental State Examination (MMSE) and results were obtained from the medical records. Neuropsychological assessments were conducted for all eligible participants using the validated Chinese version of the Mini-Mental State Examination (MMSE). Since MMSE performance is most influenced by educational level, it is strongly recommended to consider education level when interpreting MMSE results. Therefore, in this study, the educational stratification cut-off points were selected based on population-based norms in China: 17 points for the uneducated, 20 points for those educated for 1–6 years, and 24 points for those educated for more than 7 years as the cut-off for cognitive impairment (18).

### Statistical methods

First, analysis was conducted using SPSS 2 6.0 statistical software (IBM, Armonk, NY, United States). Continuous variables that conform to a normal distribution are expressed as mean ± standard deviation, and comparisons between the two groups were performed using a two-tailed Student’s *t*-test. Data that do not conform to a normal distribution are expressed as median (interquartile range) and analyzed using the Mann–Whitney *U* test. Categorical variables are expressed as numbers (percentages), and comparisons between the two groups were performed using the *χ*^2^ test. We applied a binary logistic regression model to assess the relationship between NLR and cognitive function and the severity of CSVD burden. The predictive value of the NLR level for cognitive impairment was identified by drawing receiver operating characteristic (ROC) curve. And the optimal cut-off point of serum NLR levels in patients with cognitive impairment was predicted according to the maximum value of the Youden Index. To evaluate the relationship between NLR and the total burden of CSVD, the Spearman correlation between NLR levels and CSVD total burden scores was calculated.

Secondly, we used R version 4. 4.0 (R Foundation for Statistical Computing, Vienna, Austria) to create restricted cubic spline plots and conduct mediation analysis. Using the rms Packages in R to create restricted cubic spline plots, we further explored the relationship between NLR and the risk of cognitive impairment as well as the burden of severe CSVD. Finally, we used R’s plyr and mediation Packages to determine whether the CSVD burden mediates the relationship between NLR and cognitive impairment. A simple mediation model (Model 4) was employed for the mediation analysis. The bootstrap method was used for 5,000 repeated samples to obtain more stable mediation model results.

## Results

### Participant characteristics

A total of 148 patients (median age: 68 years, interquartile range: 60–72 years; 100 males and 48 females) participated in the current analysis. According to MMSE scores and years of education, the cognitive impairment group included 89 patients, while the no cognitive impairment group included 59 patients. Table 1 provides detailed information about the two groups. Compared to patients without cognitive impairment, those with cognitive impairment had higher Hcy levels (*p* < 0.05). There were statistically significant differences in NLR, MLR, SII, and total CSVD burden scores between the two groups (*p* < 0.05), while PLR showed no significant difference. There were no statistically significant differences in the remaining demographic and general clinical data between the groups.

**Table 1:** Characteristics of the participants between cognitive impairment group and no cognitive impairment group.

Clinical characteristics	Cognitive impairment group (n = 89)	NO cognitive impairment group (*n* = 59)	*p*-value
Age, median (IQR), years	68(59.5,74.0)	68(60.0,71.0)	0.906
Sex, female, *n* (%)	25(28.1)	23(39.0)	0.166
BMI, median (IQR), kg/m2	25.25(23.01,27.73)	25.24(24.14,28.34)	0.308
Diabetes, *n* (%)	30(33.7)	17(28.8)	0.531
Hypertension, *n* (%)	63(70.8)	39(66.1)	0.547
Coronary heart disease, *n* (%)	7(7.9)	6(10.2)	0.628
History of stroke, *n* (%)	72(80.9)	45(76.3)	0.498
Current smoking, *n* (%)	19(21.3)	14(23.7)	0.733
Alcohol use, *n* (%)	7(7.9)	10(16.9)	0.090
Education, median (IQR), year	9(8,12)	9(6,12)	0.638
TC, mean (SD), mmol/L	4.26 ± 1.05	4.45 ± 1.18	0.305
TG, median (IQR), mmol/L	1.10(0.80,1.55)	1.20(0.84,1.62)	0.522
HDL-C, mean (SD), mmol/L	1.13 ± 0.26	1.18 ± 0.29	0.263
LDL-C, median (SD), mmol/L	2.59 ± 0.78	2.71 ± 0.88	0.378
tHcy, median (IQR), μmol/L	15.30(12.00,20.20)	13.50(11.10,17.20)	0.045*
NLR, mean (SD)	2.63 ± 0.80	2.25 ± 0.84	0.007*
PLR, median (IQR)	128.93(105.89,164.35)	122.88(101.46,147.59)	0.365
MLR, median (IQR)	0.23(0.18,0.28)	0.18(0.16,0.25)	0.020*
SII, median (IQR)	527(403,671)	450(315,619)	0.021*
Fazekas	5(3,6)	3(2,5)	0.003*
Total CSVD burden score			0.001*
1, *n* (%)	9(10.1)	7(11.9)	
2, *n* (%)	11(12.4)	17(28.8)	
3, *n* (%)	18(20.2)	21(35.6)	
4, *n* (%)	51(57.3)	14(23.7)	

*p < 0.05.

SD, standard deviation; IQR, interquartile range; BMI, body mass index; TC, total cholesterol; TG, triglyceride; HDL-C, high density lipoprotein cholesterol; LDL-C, low density lipoprotein cholesterol; UA, uric acid; tHcy, total homocysteine; NLR, neutrophil/lymphocyte ratio; PLR, platelet/lymphocyte ratio; MLR, monocyte/lymphocyte ratio; SII, systemic immune-inflammation index; CSVD, cerebral small vessel disease.

### The relationship between NLR and cognitive impairment

**Figure 1:**
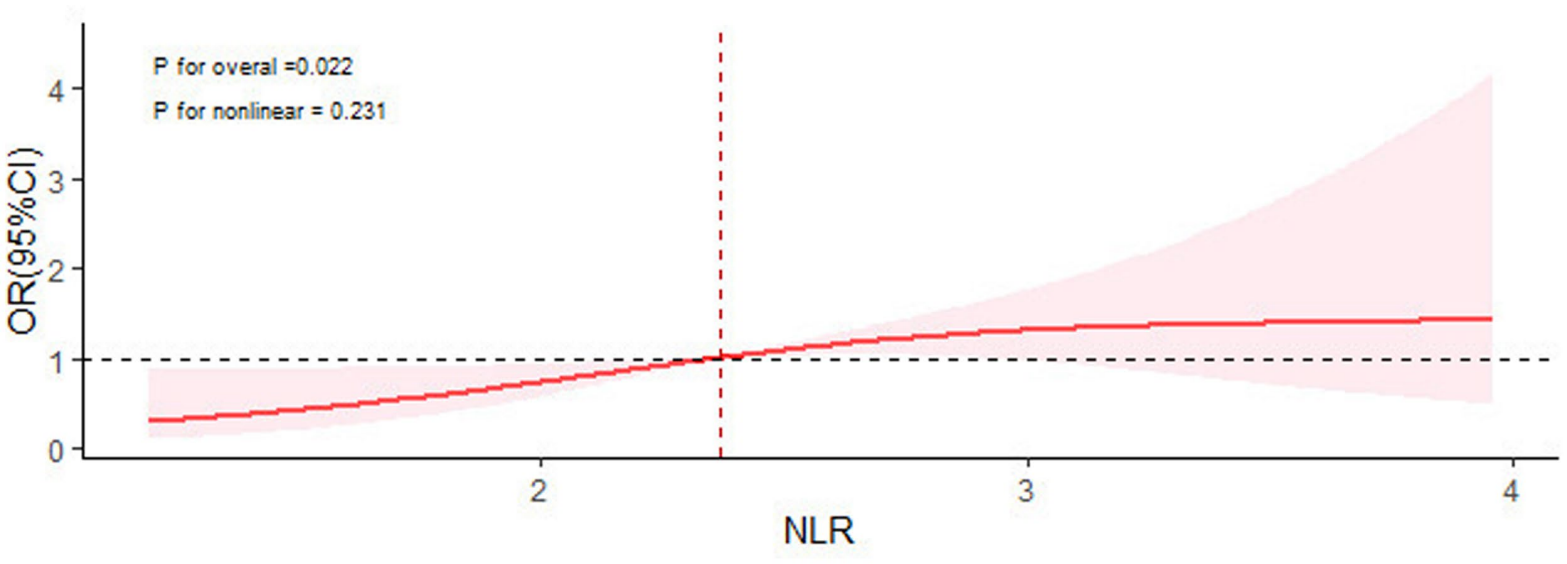
Restricted cubic spline for the association between serum NLR and the risk of cognitive impairment.

**Figure 2:**
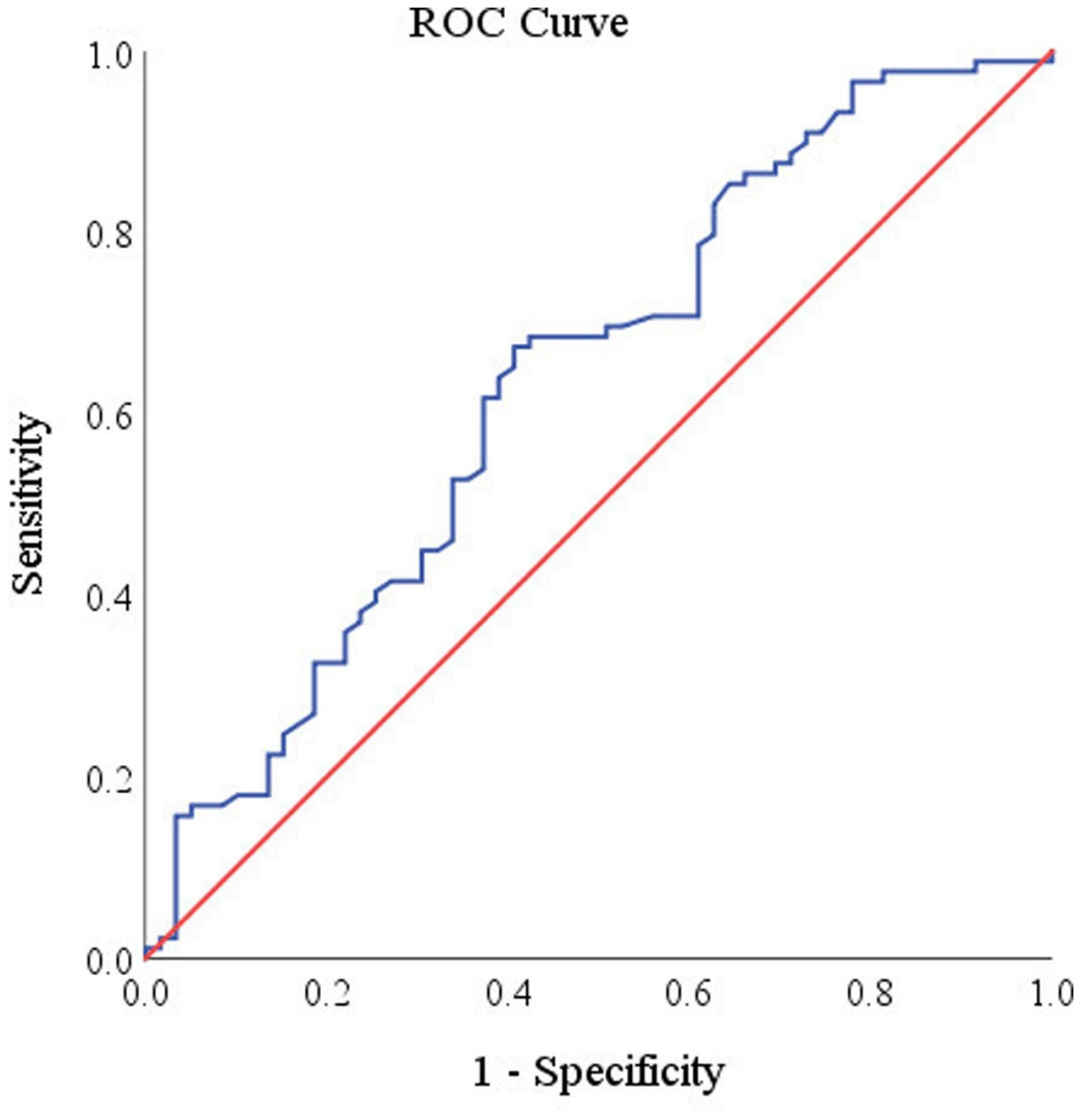
Receiver operating characteristic (ROC) curve of NLR levels for cognitive impairment. The specificity was 0.593 (1–0.407) and sensitivity was 0.674.

In this study, we used a logistic regression model to explore the relationship between NLR and cognitive impairment (Table 2). After adjusting for homocysteine and total burden of CSVD factors, the logistic regression results showed that NLR was significantly associated with the occurrence of cognitive impairment (OR: 1.625; 95% CI: 1.049 to 2.517; *p* = 0.030), while MLR and SII were not independent risk factors for the occurrence of cognitive impairment. Additionally, the results of the restricted cubic spline graph more clearly demonstrate the dose–response curve of NLR and the risk of cognitive impairment (Figure 1). After adjusting for all confounding factors, NLR exhibited a linear relationship with the risk of cognitive impairment (*P* for total = 0.022, *P* for non-linear = 0.231). It is obtained by drawing ROC curve the NLR levels of patients with cognitive impairment were optimally cut-off at 2.26 (Figure 2), with an area under the curve (AUC) of 0.636 (95% CI: 0.543 to 0.728, *p* < 0.05).

**Table 2:** The logistic regression analyses of risk factors for cognitive impairment.

Factors	Multivariable analysis	
	OR(95% CI)	*P*-value
tHcy	1.004(0.970–1.039)	0.827
CSVD burden	1.565(1.111–2.205)	0.010*
NLR	1.625(1.049–2.517)	0.030*
MLR	45.428(0.621–3325.424)	0.081
SII	1.002(1.000–1.004)	0.097

*P < 0.05.

tHcy, total homocysteine; tHcy; NLR, neutrophil/lymphocyte ratio; PLR, platelet/lymphocyte ratio; MLR, monocyte/lymphocyte ratio; SII, systemic immune-inflammation index; CSVD, cerebral small vessel disease.

### The correlation between NLR and total burden of CSVD

**Figure 3:**
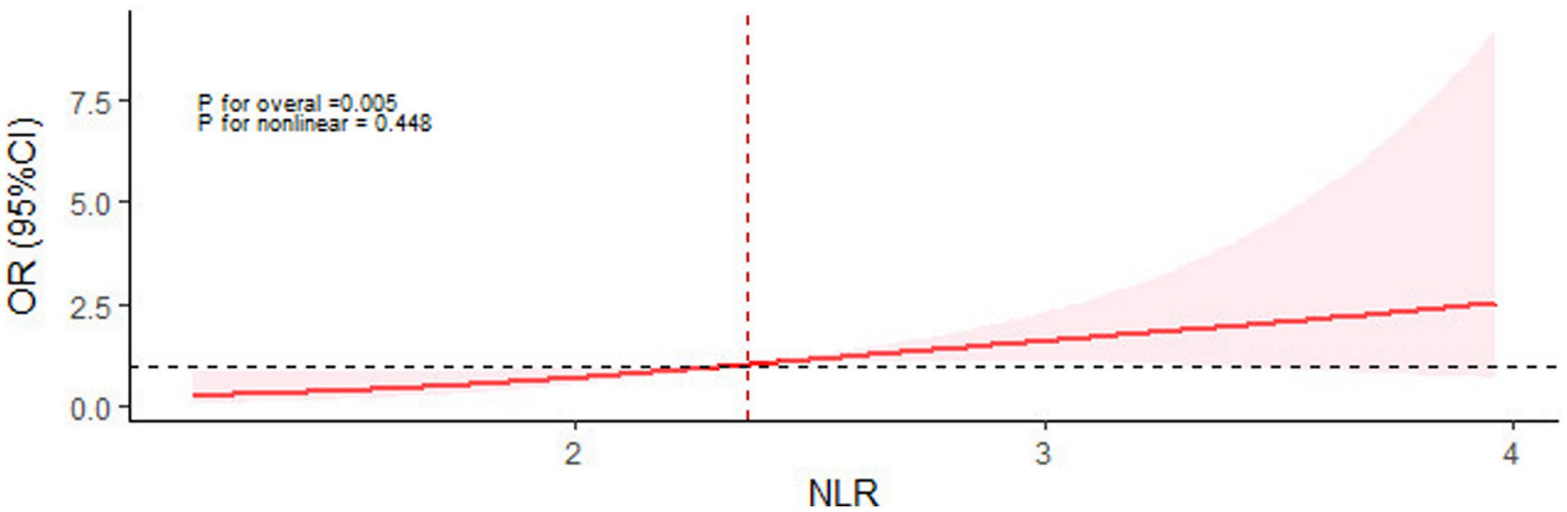
Restricted cubic spline for the association between serum NLR and the CSVD burden.

In the univariate binary logistic regression analysis, elevated NLR levels were associated with an increased risk of severe CSVD burden (OR = 1.958, 95% CI: 1.215 ~ 3.158, *p* = 0.006). Further adjustment for age, total cholesterol, and low-density lipoprotein revealed that elevated NLR levels are an independent risk factor for severe CSVD (OR = 2.246, 95% CI: 1.346 ~ 3.750, *p* = 0.002) as shown in Table 3. Similarly, we further illustrate the dose–response curve of NLR and the total burden of CSVD by plotting the results of the restricted cubic spline (Figure 3). After adjusting for all confounding factors, a linear relationship between NLR and the total burden of CSVD was observed (*P* for total = 0.005, *P* for non-linear = 0.448). In addition, a Spearman correlation analysis between NLR levels and CSVD total burden scores (Table 4) showed a positive correlation (rs = 0.246, *p* = 0.003), indicating that higher NLR levels are associated with more severe CSVD.

**Table 3:** The logistic regression analysis of risk factors for severe CSVD burden.

Factors	Univariable analysis		Multivariable analysis^a^	
	OR(95% CI)	*P*-value	OR(95% CI)	*P*-value
Age	1.067(1.025,1.112)	0.002*	1.081(1.029,1.135)	0.002*
Sex	1.708(0.818,3.564)	0.154	—	—
Education	0.990(0.893,1.096)	0.842	—	—
BMI	0.920(0.832,1.-16)	0.100	—	—
Diabetes	0.743(0.353,1.564)	0.434	—	—
Hypertension	1.410(0.668,2.974)	0.367	—	—
History of stroke	1.162(0.496,2.726)	0.729	—	—
Coronary heart disease	0.324(0.102,1.026)	0.055	—	—
Current smoking	0.805(0.351,1.844)	0.608	—	—
Alcohol use	0.426(0.153,1.190)	0.104	—	—
TC	0.466(0.320,0.679)	<0.001*	1.124(0.323,3.906)	0.855
TG	0.944(0.623,1.429)	0.786	—	—
LDL-C	0.351(0.213,0.578)	<0.001*	0.279(0.052,1.491)	0.136
HDL-C	0.377(0.102,1.394)	0.144	—	—
tHcy	1.045(0.933,1.099)	0.091	—	—
NLR	1.958(1.215,3.158)	0.006*	2.246(1.346,3.750)	0.002*
PLR	1.004(0.995,1.012)	0.374	—	—
MLR	45.994(0.397,5327.384)	0.114	—	—
SII	1.002(1.000,1.003)	0.132	—	—

*P < 0.05. ^a^Adjusted with *P* < 0.05 for age, TC, LDL-C, and NLR.

BMI, body mass index; TC, total cholesterol; TG, triglyceride; HDL-C, high density lipoprotein cholesterol; LDL-C, low density lipoprotein cholesterol; tHcy, total homocysteine; NLR, neutrophil/lymphocyte ratio; PLR, platelet/lymphocyte ratio; MLR, monocyte/lymphocyte ratio; SII, systemic immune-inflammation index.

**Table 4:** Spearman correlation analysis of NLR and total CSVD burden score.

Factors	rs	*P*-value
CSVD burden score	0.246	0.003*

*p < 0.05.

CSVD, cerebral small vessel disease.

### The mediating role of CSVD total burden

**Figure 4:**
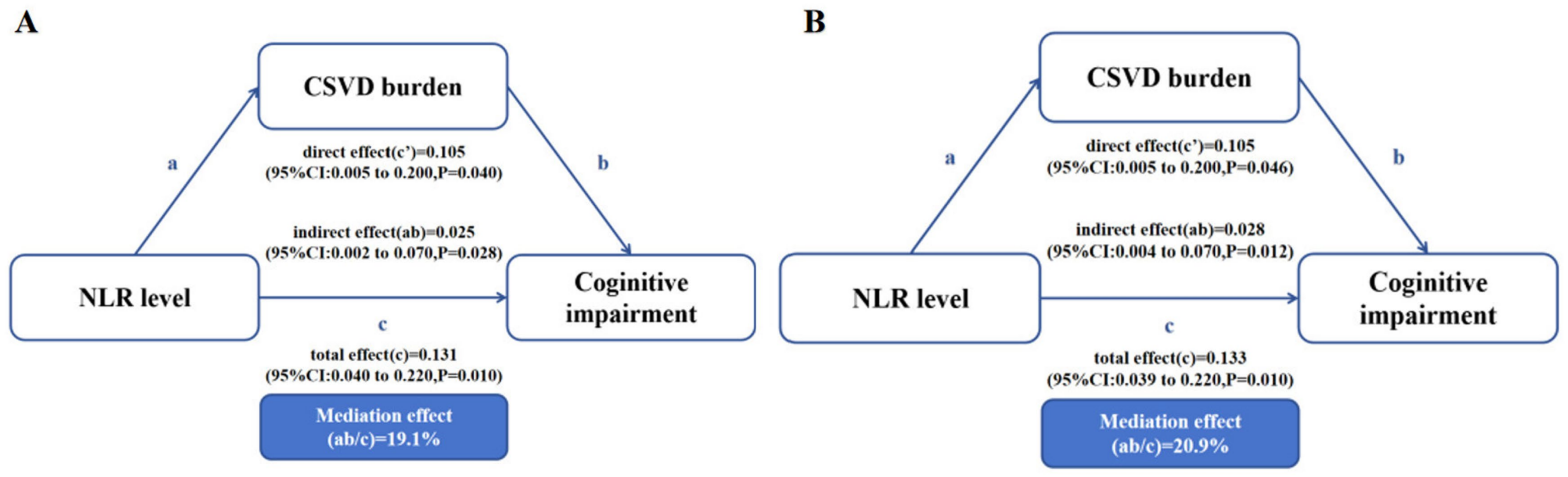
Mediation analysis is shown for the presence of severe CSVD burden as a mediator in the relation between NLR and cognitive impairment. **(A)** Unadjusted. **(B)** Adjusted for age, TC, LDL-C, and tHcy.

This section of the study employs a mediation model to explore whether the total burden of CSVD mediates the risk of cognitive impairment associated with increased NLR. Results of the mediation analyses are presented in Figure 4. There is a significant total effect (c) and direct effect (c’) of NLR on cognitive impairment (*p* < 0.05), and when the severe CSVD total burden score is included in the model, there is a significant indirect effect (ab = 0.025, 95% CI: 0.002 to 0.070, *p* = 0.028). After adjusting for age, TC, LDL-C, and tHcy, the mediation effect of CSVD total burden still exists (ab = 0.028, 95% CI: 0.004 to 0.070, *p* = 0.012). After adjusting for confounding factors, 20.9% of the total effect of NLR on cognitive impairment can be attributed to the presence of CSVD burden (Figure 4).

## Discussion

In this study, we found that NLR is an independent risk factor for the occurrence of cognitive impairment and severe CSVD burden in patients. In patients with CSVD, as NLR increases, the risk of cognitive impairment and the severity of the total CSVD burden also increase. Furthermore, the results of the mediation model suggest that the effect of NLR on the occurrence of cognitive impairment is mediated by the total burden of CSVD, meaning that an increase in NLR may exacerbate the severity of CSVD, thereby increasing the risk of cognitive impairment.

Currently, several studies have explored the relationship between NLR and cognitive impairment in different populations. A meta-analysis found that compared to healthy individuals, the NLR was significantly elevated in patients with mild cognitive impairment and Alzheimer’s disease (19). Additionally, a prospective study on Alzheimer’s disease also confirmed the above viewpoint. However, the study found no significant difference in NLR levels between aMCI patients and AD patients, suggesting that NLR cannot predict the transition of patients from mild cognitive impairment to Alzheimer disease (20). But a prospective study on amnestic mild cognitive impairment found that, at the end of follow-up, the NLR of patients who progressed to dementia from those who remained in mild cognitive impairment was significantly elevated (21). There are also studies (22) exploring the correlation between NLR and cognitive, neuroimaging, and pathological aspects in Alzheimer’s disease patients, finding that elevated NLR is significantly associated with total cognitive function, memory function, and executive ability levels in patients. Moreover, elevated NLR is associated with increased levels of *β*-amyloid and total T-tau protein in the cerebrospinal fluid of Alzheimer’s disease patients. In terms of neuroimaging, elevated NLR is associated with reduced hippocampal volume and decreased thickness of the entorhinal cortex. The above study suggests that NLR may mediate the cognitive imaging in Alzheimer’s disease patients through Aβ and T-tau. In a follow-up study on acute ischemic stroke, it was found that NLR, as a categorical variable, is an independent risk factor for post-stroke cognitive impairment, with patients in the higher NLR group showing significantly poorer total cognitive function (23, 24). A study using brain FDG FET to predict post-stroke cognitive impairment found that higher metabolic cognitive feature scores in FDG FET of stroke patients were positively correlated with NLR. Moreover, the predictive model for post-stroke cognitive impairment is more accurate when NLR is considered as an auxiliary factor (25). Therefore, we have reason to believe that NLR, as a peripheral blood inflammatory marker, is a feasible tool for assessing cognitive impairment.

An increasing number of research findings indicate that NLR is associated with the development of different imaging characteristics in CSVD patients, which is also very important for the occurrence of cognitive impairment. A study conducted on a community population found that higher NLR levels were significantly associated with moderate to severe basal ganglia EPVS (26). Interestingly, some studies suggest that NLR may also affect the brain electrical activity of CSVD patients through imaging, thereby leading to or exacerbating cognitive impairment (27). Compared to previous studies, the innovation of this research lies in the discovery of a significant mediating effect of the total burden of CSVD on the relationship between NLR and cognitive impairment. It is worth noting that previous studies have found a common mechanism between severe total burden of CSVD and the occurrence of cognitive impairment (28), including endothelial dysfunction, blood–brain barrier disruption, oxidative stress response, and chronic neuroinflammation (29–32). Therefore, when studying the relationship between NLR and cognitive impairment, the severity of CSVD should be considered. In our study, it was confirmed that the elevated NLR levels in CSVD patients may exacerbate the progression of CSVD, thereby increasing the risk of cognitive impairment.

Although we have some new findings regarding the relationship between NLR, CSVD, and cognitive impairment, there are still some limitations: first, this study is a retrospective study and cannot confirm the causality between the variables. Although the results of the mediation analysis support the hypothesis that the burden of CSVD mediates, to some extent, the association between NLR in CSVD patients and the occurrence of cognitive impairment, further prospective studies are needed to confirm this. Second, this is a study with a small sample size, which may lead to selection bias. For example, in a large sample study, gender was found to have an effect on the prognosis of patients with cerebrovascular disease (33), which was not found in this study. Third, the severity of cognitive impairment was not categorized; the MMSE scale was used for cognitive assessment, which did not allow for a detailed evaluation of cognitive domains. Therefore, we will expand the sample size to verify the effect of relevant risk factors on outcomes and use broader neuropsychological testings to explore the relationship between NLR and different cognitive domains in future studies. Furthermore, we will conduct prospective studies to explore the causal relationship between variables.

## Conclusion

In summary, our study indicates that in CSVD patients, NLR is associated with the total burden severity and cognitive impairment. The mediation analysis results show that an increase in NLR raises the risk of cognitive impairment, partly due to its impact on the severity of the total burden in CSVD. However, the causal relationship of this association needs to be further established through prospective studies.

## Data Availability

The original contributions presented in the study are included in the article/supplementary material, further inquiries can be directed to the corresponding author.

## References

[1] Livingston G Sommerlad A Orgeta V Costafreda SG Huntley J Ames D. Dementia prevention, intervention, and care. Lancet. (2017) 390:2673–734. doi: 10.1016/S0140-6736(17)31363-6, PMID: 28735855

[2] Rundek T Tolea M Ariko T Fagerli EA Camargo CJ. Vascular Cognitive Impairment (VCI). Neurotherapeutics. (2022) 19:68–88. doi: 10.1007/s13311-021-01170-y, PMID: 34939171 PMC9130444

[3] Litak J Mazurek M Kulesza B Szmygin P Litak J Kamieniak P. Cerebral Small Vessel Disease. Int J Mol Sci. (2020) 21:21. doi: 10.3390/ijms21249729, PMID: 33419271 PMC7766314

[4] Wardlaw JM Smith EE Biessels GJ Cordonnier C Fazekas F Frayne R. Neuroimaging standards for research into small vessel disease and its contribution to ageing and neurodegeneration. Lancet Neurol. (2013) 12:822–38. doi: 10.1016/S1474-4422(13)70124-8, PMID: 23867200 PMC3714437

[5] Arboix A Massons J García-Eroles L Targa C Comes E Parra O. Clinical predictors of lacunar syndrome not due to lacunar infarction. BMC Neurol. (2010) 10:31. doi: 10.1186/1471-2377-10-31, PMID: 20482763 PMC2877662

[6] Mosca M Nigro MC Pagani R De Giglio A Di Federico A. Neutrophil-to-Lymphocyte Ratio (NLR) in NSCLC, Gastrointestinal, and Other Solid Tumors: Immunotherapy and Beyond. Biomol Ther. (2023) 13:13. doi: 10.3390/biom13121803, PMID: 38136673 PMC10741961

[7] Song Q Xu SX Wu JZ Ling L Wang S Shu XH. The preoperative platelet to neutrophil ratio and lymphocyte to monocyte ratio are superior prognostic indicators compared with other inflammatory biomarkers in ovarian cancer. Front Immunol. (2023) 14:1177403. doi: 10.3389/fimmu.2023.1177403, PMID: 37457691 PMC10347525

[8] Hou Y Li X Yang Y Shi H Wang S Gao M. Serum cytokines and neutrophil-to-lymphocyte ratio as predictive biomarkers of benefit from PD-1 inhibitors in gastric cancer. Front Immunol. (2023) 14:1274431. doi: 10.3389/fimmu.2023.1274431, PMID: 38022654 PMC10643875

[9] Gong P Liu Y Gong Y Chen G Zhang X Wang S. The association of neutrophil to lymphocyte ratio, platelet to lymphocyte ratio, and lymphocyte to monocyte ratio with post-thrombolysis early neurological outcomes in patients with acute ischemic stroke. J Neuroinflammation. (2021) 18:51. doi: 10.1186/s12974-021-02090-6, PMID: 33610168 PMC7896410

[10] Zhang X Wei R Wang X Zhang W Li M Ni T. The neutrophil-to-lymphocyte ratio is associated with all-cause and cardiovascular mortality among individuals with hypertension. Cardiovasc Diabetol. (2024) 23:117. doi: 10.1186/s12933-024-02191-5, PMID: 38566082 PMC10985955

[11] Hou L Zhang S Qi D Jia T Wang H Zhang W. Correlation between neutrophil/lymphocyte ratio and cognitive impairment in cerebral small vessel disease patients: A retrospective study. Front Neurol. (2022) 13:925218. doi: 10.3389/fneur.2022.925218, PMID: 35989913 PMC9391025

[12] Peng D. Clinical practice guideline for cognitive impairment of cerebral small vessel disease. Aging Med. (2019) 2:64–73. doi: 10.1002/agm2.12073, PMID: 31942514 PMC6880706

[13] Fazekas F Kleinert R Offenbacher H Schmidt R Kleinert G Payer F. Pathologic correlates of incidental MRI white matter signal hyperintensities. Neurology. (1993) 43:1683–9. doi: 10.1212/WNL.43.9.1683, PMID: 8414012

[14] Doubal FN MacLullich AM Ferguson KJ Dennis MS Wardlaw JM. Enlarged perivascular spaces on MRI are a feature of cerebral small vessel disease. Stroke. (2010) 41:450–4. doi: 10.1161/STROKEAHA.109.564914 20056930

[15] Gregoire SM Chaudhary UJ Brown MM Yousry TA Kallis C Jäger HR. The Microbleed Anatomical Rating Scale (MARS): reliability of a tool to map brain microbleeds. Neurology. (2009) 73:1759–66. doi: 10.1212/WNL.0b013e3181c34a7d 19933977

[16] Staals J Makin SD Doubal FN Dennis MS Wardlaw JM. Stroke subtype, vascular risk factors, and total MRI brain small-vessel disease burden. Neurology. (2014) 83:1228–34. doi: 10.1212/WNL.0000000000000837, PMID: 25165388 PMC4180484

[17] Kim JM Park KY Kim HR Ahn HY Pantoni L Park MS. Association of Bone Mineral Density to Cerebral Small Vessel Disease Burden. Neurology. (2021) 96:e1290–300. doi: 10.1212/WNL.0000000000011526 33431517

[18] Li H Jia J Yang Z. Mini-Mental State Examination in Elderly Chinese: A Population-Based Normative Study. J Alzheimers Dis. (2016) 53:487–96. doi: 10.3233/JAD-160119, PMID: 27163822

[19] Huang LT Zhang CP Wang YB Wang JH. Association of Peripheral Blood Cell Profile With Alzheimer's Disease: a meta-analysis. Front Aging Neurosci. (2022) 14:888946. doi: 10.3389/fnagi.2022.888946, PMID: 35601620 PMC9120416

[20] Cervellati C Pedrini D Pirro P Guindani P Renzini C Brombo G. Neutrophil-Lymphocytes Ratio as Potential Early Marker for Alzheimer's Disease. Mediat Inflamm. (2024) 2024:6640130. doi: 10.1155/2024/6640130, PMID: 38974600 PMC11227945

[21] Tondo G Aprile D De Marchi F Sarasso B Serra P Borasio G. Investigating the Prognostic Role of Peripheral Inflammatory Markers in Mild Cognitive Impairment. J Clin Med. (2023) 12:12. doi: 10.3390/jcm12134298, PMID: 37445333 PMC10342872

[22] Hou JH Ou YN Xu W Zhang PF Tan L Yu JT. Association of peripheral immunity with cognition, neuroimaging, and Alzheimer's pathology. Alzheimers Res Ther. (2022) 14:29. doi: 10.1186/s13195-022-00968-y, PMID: 35139899 PMC8830026

[23] Lee M Lim JS Kim CH Lee SH Kim Y Hun Lee J. High Neutrophil-Lymphocyte Ratio Predicts Post-stroke Cognitive Impairment in Acute Ischemic Stroke Patients. Front Neurol. (2021) 12:693318. doi: 10.3389/fneur.2021.693318, PMID: 34276542 PMC8280279

[24] Shang T Ma B Shen Y Wei C Wang Z Zhai W. High neutrophil percentage and neutrophil-lymphocyte ratio in acute phase of ischemic stroke predict cognitive impairment: A single-center retrospective study in China. Front Neurol. (2022) 13:907486. doi: 10.3389/fneur.2022.907486, PMID: 36071906 PMC9441903

[25] Lee R Choi H Park KY Kim JM Seok JW. Prediction of post-stroke cognitive impairment using brain FDG PET: deep learning-based approach. Eur J Nucl Med Mol Imaging. (2022) 49:1254–62. doi: 10.1007/s00259-021-05556-0, PMID: 34599654

[26] Jiang L Cai X Yao D Jing J Mei L Yang Y. Association of inflammatory markers with cerebral small vessel disease in community-based population. J Neuroinflammation. (2022) 19:106. doi: 10.1186/s12974-022-02468-0, PMID: 35513834 PMC9072153

[27] Guo X Liu Z Yuan W Wei A Luo G. Electroencephalographic power spectrum changes in cerebral small vessel disease combined with cognitive dysfunction and its relationship with neutrophil/lymphocyte ratio and its clinical value – a pilot study. Front Neurol. (2023) 14:1300240. doi: 10.3389/fneur.2023.1300240, PMID: 38283675 PMC10820721

[28] Hamilton OKL Backhouse EV Janssen E Jochems ACC Maher C Ritakari TE. Cognitive impairment in sporadic cerebral small vessel disease: a systematic review and meta-analysis. Alzheimers Dement. (2021) 17:665–85. doi: 10.1002/alz.12221, PMID: 33185327 PMC8593445

[29] Hughes CG Patel MB Brummel NE Thompson JL McNeil JB Pandharipande PP. Relationships between markers of neurologic and endothelial injury during critical illness and long-term cognitive impairment and disability. Intensive Care Med. (2018) 44:345–55. doi: 10.1007/s00134-018-5120-1, PMID: 29523900 PMC5870884

[30] Inoue Y Shue F Bu G Kanekiyo T. Pathophysiology and probable etiology of cerebral small vessel disease in vascular dementia and Alzheimer's disease. Mol Neurodegener. (2023) 18:46. doi: 10.1186/s13024-023-00640-5, PMID: 37434208 PMC10334598

[31] Rajeev V Fann DY Dinh QN Kim HA De Silva TM Lai MKP. Pathophysiology of blood brain barrier dysfunction during chronic cerebral hypoperfusion in vascular cognitive impairment. Theranostics. (2022) 12:1639–58. doi: 10.7150/thno.68304, PMID: 35198062 PMC8825579

[32] Wan S Dandu C Han G Guo Y Ding Y Song H. Plasma inflammatory biomarkers in cerebral small vessel disease: a review. CNS Neurosci Ther. (2023) 29:498–515. doi: 10.1111/cns.14047, PMID: 36478511 PMC9873530

[33] Inogés M Arboix A García-Eroles L Sánchez-López MJ. Gender predicts differences in acute ischemic cardioembolic stroke profile: emphasis on woman-specific clinical data and early outcome-the experience of Sagrat Cor Hospital of Barcelona Stroke Registry. Medicina. (2024) 60:60. doi: 10.3390/medicina60010101, PMID: 38256361 PMC10819324

